# The Prognostic Value of Proliferative Activity in Cutaneous Melanoma: A Pilot Study Evaluating the Mitotic Rate and Ki67 Index to Predict Patient Outcomes

**DOI:** 10.3390/biomedicines12061318

**Published:** 2024-06-13

**Authors:** Dana Antonia Tapoi, Ancuța-Augustina Gheorghișan-Gălățeanu, Laura Maria Gosman, Diana Derewicz, Mariana Costache

**Affiliations:** 1 Department of Pathology, Carol Davila University of Medicine and Pharmacy, 020021 Bucharest, Romania; dana-antonia.tapoi@drd.umfcd.ro (D.A.T.); mariana.costache@umfcd.ro (M.C.); 2 Department of Pathology, University Emergency Hospital, 050098 Bucharest, Romania; 3 Doctoral School, Carol Davila University of Medicine and Pharmacy, 020021 Bucharest, Romania; ancuta.gheorghisan@umfcd.ro; 4 Department of Pathology, Saint Pantelimon Clinical Emergency Hospital, 021659 Bucharest, Romania; 5 Department of Pediatrics, Carol Davila University of Medicine and Pharmacy, 020021 Bucharest, Romania; diana.derewicz@umfcd.ro; 6 Department of Pediatric Hematology and Oncology, Marie Sklodowska Curie Clinical Emergency Hospital, 041447 Bucharest, Romania

**Keywords:** cutaneous melanoma, mitotic rate, Ki67, perineural invasion, prognosis

## Abstract

Proliferative activity in cutaneous melanomas can be appreciated both histopathologically by counting mitotic figures and immunohistochemically through the Ki67 index, but the prognostic value of each method is still a matter of debate. In this context, we performed a retrospective study on 33 patients diagnosed with cutaneous melanomas between 2013 and 2018 in order to evaluate progression-free survival and overall survival. Multivariate Cox proportional hazards regression was performed by considering both clinical histopathological and immunohistochemical features. The mitotic rate was significantly independently associated with both outcomes, while the Ki67 index was not an independent prognostic factor. However, the Ki67 predictive accuracy could be improved by establishing both a cut-off value and a standardized protocol for evaluating its expression. Until these desiderata are met, the mitotic rate remains superior to the Ki67 index for predicting prognosis in cutaneous melanomas, as also has the advantage of being easily interpreted in a standard histopathological examination regardless of the pathologist’s experience and with no further financial expenses. Importantly, this is one of very few articles that has shown perineural invasion to be an independent prognostic factor for both progression-free survival and overall survival in cutaneous melanomas. As a consequence, this parameter should become a mandatory feature in the histopathological evaluation of cutaneous melanomas as it can improve the identification of patients who are at high risk for disease progression.

## 1. Introduction

Cutaneous melanomas represent highly aggressive malignancies, causing the vast majority of skin-cancer-related deaths, particularly in advanced stages [1]. In this context, the Breslow depth of invasion is the most important prognostic factor as it is used, together with epidermal ulceration, to stage cutaneous melanomas [2]. Nevertheless, locally advanced tumors (pT3 and pT4) often display unpredictable behaviors and require additional prognostic factors to identify high-risk patients in order to provide the best therapeutic strategies. As a consequence, various clinical and histopathological parameters, such as age, ulceration, lympho-vascular invasion, and mitotic counts [3,4,5,6,7], have been shown to predict prognosis in cutaneous melanomas [3,4,5,6,7].

An immunohistochemical analysis is a widely used method in evaluating cutaneous melanocytic neoplasms, particularly for diagnosing undifferentiated or dedifferentiated tumors or for distinguishing between severely dysplastic nevi and melanomas. Nevertheless, an immunohistochemical analysis has a limited prognostic value in such cases [8,9,10]. Among the possible predictive markers, the proliferation marker Ki67 requires special attention as it has a proven prognostic significance in certain other malignancies such as breast carcinomas or neuroendocrine neoplasms [11,12]. The Ki67 antibody was named after the city of Kiel, where it was discovered, and the number of the original clone, and it is a non-histone nuclear protein that is expressed during cell division [12].

From the perspective of skin cancers, the Ki67 index has been associated with the prognosis of Merkel cell carcinomas, a category of neuroendocrine tumors, in univariate analysis, but not in multivariate analysis [13]. Additionally, Ki67 has been shown to be more useful than mitotic counts for predicting prognosis in Merkel cell carcinomas [14]. Nevertheless, despite having promising results, the predictive significance depends on the adopted cut-off value, and it therefore varies based on the study [15,16,17]. Similarly, the predictive value of this parameter is uncertain in the case of cutaneous melanomas [18,19,20,21]. However, according to the guidelines developed by the AJCC [2], the proliferative activity of melanomas should be assessed by reporting the mitotic index, which is considered to have prognostic value [22,23]. Therefore, this research aimed to evaluate the association between the Ki67 index expression and progression-free survival and overall survival of patients with pT3 and pT4 cutaneous melanomas. Additionally, the correlation between Ki67 index values and the number of mitoses/mm^2^ was analyzed to determine the extent to which these two parameters, which evaluate proliferative activity, are equivalent. Finally, the correlation between the Breslow depth and Ki67 expression and the correlation between the Breslow depth and the number of mitoses/mm^2^ were also analyzed to determine whether proliferative activity is correlated with invasion depth. This study only evaluated patients with pT3 and pT4 cutaneous melanomas as such cases are the most likely to have an aggressive course yet remain rather heterogenous in terms of behavior and may display unpredictable evolutions. As a consequence, these melanomas require complex follow-up and therapies in contrast to less invasive melanomas, which may be cured by surgery alone without further treatment. In this context, a better characterization of histopathological and immunohistochemical prognostic factors is expected to significantly improve the management of patients.

## 2. Materials and Methods

We conducted a retrospective study that analyzed 33 patients diagnosed with cutaneous melanomas in stages pT3 and pT4, between 2012 and 2017, at the University Emergency Hospital of Bucharest in Romania. The inclusion criteria for the study were as follows:

- A histopathologically confirmed diagnosis of cutaneous melanoma with a Breslow index between 2 and 4 mm (stage pT3) or over 4 mm (stage pT4), regardless of lymph node invasion;
- The performance of imaging investigations (cranial, thoracic, and abdominopelvic computerized tomography) immediately after the initial diagnosis to detect possible metastatic lesions;
- Patient follow-up for a minimum of 5 years or until the patient’s death to evaluate progression-free survival (PFS) or overall survival (OS).

This study was approved by the Ethics Committee at the University Emergency Hospital of Bucharest, Romania. This study followed the principles of the Helsinki Declaration. Additionally, every patient signed an informed consent form in order to be included in this research.

Histopathological processing of specimens was carried out according to standard protocols for formalin-fixed, paraffin-embedded tissue sections. The histopathological parameters evaluated were as follows:

- Invasion depth measured in millimeters (Breslow index);
- Number of mitoses/mm^2^;
- Others: melanoma subtype, the presence of regression, ulceration, microsatelliets, tumor necrosis, lympho-vascular invasion, and perineural invasion.

The mitotic counts were evaluated using the hot-stop method, which implied scanning all the slides at a 10× objective magnification in order to identify the area with the most intense mitotic activity. Hence, the mitotic figures in that spot were counted at a 40× objective magnification, and the count was extended to adjacent non-overlapping fields until 1 mm^2^ was assessed. Additionally, immunohistochemical staining for the proliferation marker Ki67 was performed using a rabbit monoclonal antibody provided by Biocare, clone SP6. The value of the Ki67 index for each case was determined as follows:

- Evaluation of the entire section overall at a 10× objective magnification to identify areas with the highest proliferative activity;
- Five photographs taken of the areas with the highest proliferative activity at a 40× objective magnification;
- Printing of the photos and counting 100 cells within each;
- Determination of the number of Ki67-positive cells out of every 100 cells counted;
- For a cell to be considered positive, it must exhibit intense nuclear Ki67 expression;
- Calculation of the mean Ki67 index by summing the values obtained for each photo and dividing by 5.

Two pathologists (D.A.Ț. and L.M.G.) individually performed these analyses, and the differences in results were settled by consulting a third pathologist (M.C.).

Descriptive statistics, including the mean, standard deviation (SD), median, minimum and maximum values, and 95% confidence intervals (CI) were performed for continuous variables. Univariate and multivariate Cox proportional hazards regression analyses were performed on the mitotic counts and Ki67 index, using PFS and OS as the outcomes. Multivariate analyses were also performed for patients’ age and gender, as well as the other histopathological prognostic factors above. This method provided hazard ratios (HR) with 95%CI and *p*-values, and a *p*-value < 0.05 was regarded as significant. The *p*-value represents the probability of an encountered difference between two groups due to chance. The level of correlation between the Ki67 index and the number of mitoses or the Breslow index was evaluated using the Spearman correlation test, and a result was considered statistically significant at a *p*-value of <0.05. The Bland–Altman plot was used to analyze the agreement between the Ki67 index value provided by the two pathologists by plotting the difference between the measurements against their average. The Kaplan–Meier product limited method was used to estimate survival probabilities, and log-rank test comparisons were made. GraphPad Prism 10.2.3 (Graphpad Software Inc., San Diego, CA, USA) was used to perform the statistical analyses.

## 3. Results

The mean age of the whole study population was 62.97 years (minimum value = 24; maximum value = 86). During the follow-up, 21 patients developed metastases, and 17 of them died, while 12 patients were free of disease progression. The mean age of the patients in the PFS group was 54.75 years (minimum = 24; maximum = 78) while the mean age of the patients in the metastatic group was 67.67 years (minimum = 40; maximum = 86), and the mean age of the patients who died during the follow-up was 69.88 years (minimum = 41; maximum = 86).

Concerning the gender of all of the patients, males and females were almost equally affected, with the male-to-female ratio being 1:1.06 (16 male patients and 17 female patients). However, in patients with progression-free survival, only four patients were male and eight were female (M:F = 1:2). On the contrary, in the metastatic group, 12 patients were male and 9 patients were female (M:F = 1.33:1). Similarly, among patients who died during the follow-up, 11 were male and 6 were female (M:F = 1.83:1).

**Figure 1:**
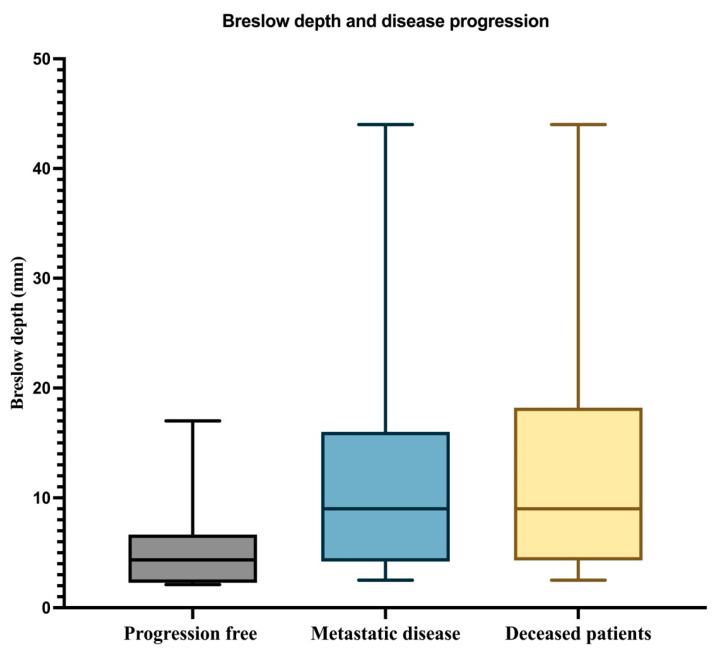
The median, minimum, and maximum values of the Breslow index in patients with PFS, patients with metastatic disease, and deceased patients.

The next step was the analysis of the Breslow depth of invasion. The median value of the Breslow index for all 33 patients analyzed was 5.6 mm. The minimum value was 2.1 mm, and the maximum value was 44 mm. For patients without progressive disease, the median value was 4.35 mm (minimum value = 2.1 mm; maximum value = 17 mm). Regarding both the patients who developed metastases and those who died during the follow-up, the median was 9 mm with a minimum value of 2.5 mm, and the maximum value was 44 mm. These data are graphically represented in Figure 1.

**Figure 2:**
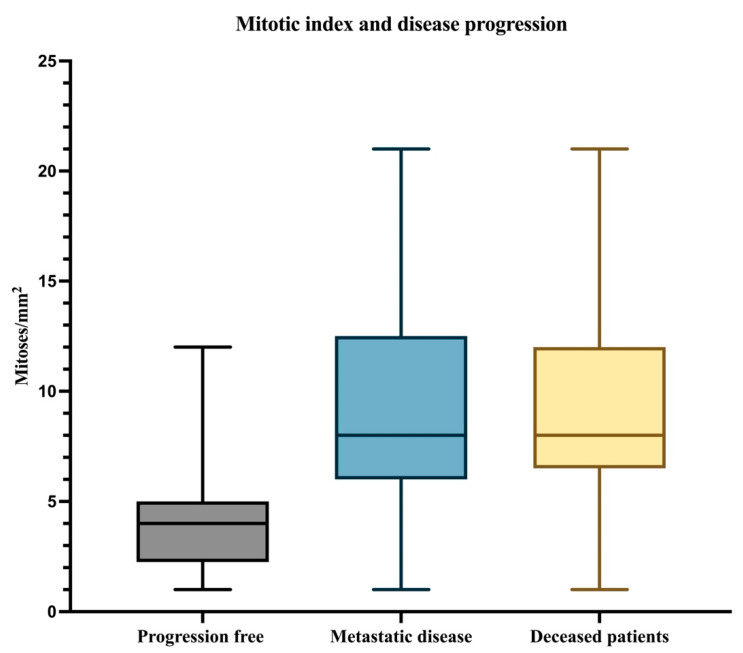
The median, minimum, and maximum values of the mitotic index in patients with PFS, patients with metastatic disease, and deceased patients.

Subsequently, the number of mitoses/mm^2^ was analyzed. It can be observed that the median mitotic index for all 33 patients was 6 mitoses/mm^2^, with a minimum value of 1 mitosis/mm^2^ and a maximum value of 21 mitoses/mm^2^. For patients without progressive disease, the median mitotic index was 4 mitoses/mm^2^, with a minimum value of 1 mitosis/mm^2^ and a maximum value of 12 mitoses/mm^2^. Regarding the patients who developed metastases and those who died, the median, minimum value, and maximum value were identical at 8 mitoses/mm^2^, 1 mitosis/mm^2^, and 21 mitoses/mm^2^, respectively (Figure 2).

In this context, through a univariate Cox analysis, the mitotic index was found to be significantly associated with overall survival but not with disease-free survival (Table 1).

**Figure 3:**
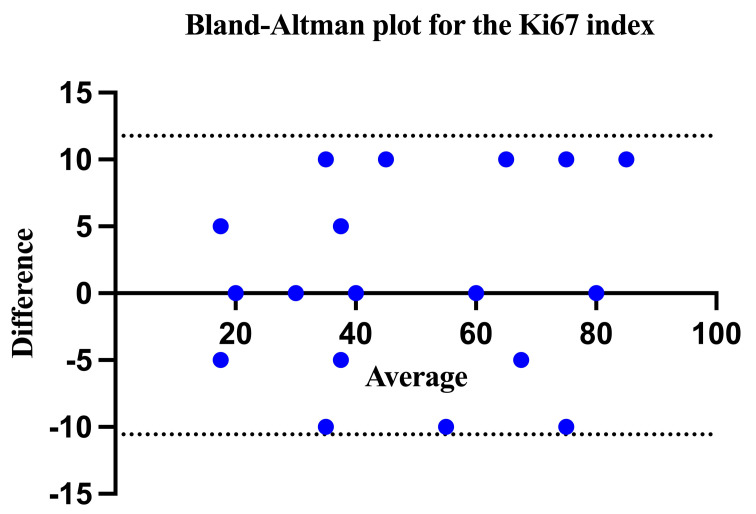
The Bland-Altman plot demonstrated a good inter-observer level of agreement in evaluating the Ki67 index.

From an immunohistochemical perspective, we analyzed the Ki67 proliferation index for all 33 patients. In this context, the first step was to determine the level of agreement between the Ki67 values provided by the two pathologists. The bias of 0.6061 (SD = 5.695) indicates a small average difference between the two measurements, suggesting that there is no significant systematic difference between the two pathologists at the average level. The 95% limits of agreement range from −10.56 to 11.77. This range indicates that 95% of the differences between the two measurements fall within these limits. However, this relatively broad range reflects a certain, yet largely acceptable, level of variability across individual measurements (Figure 3).

**Figure 4:**
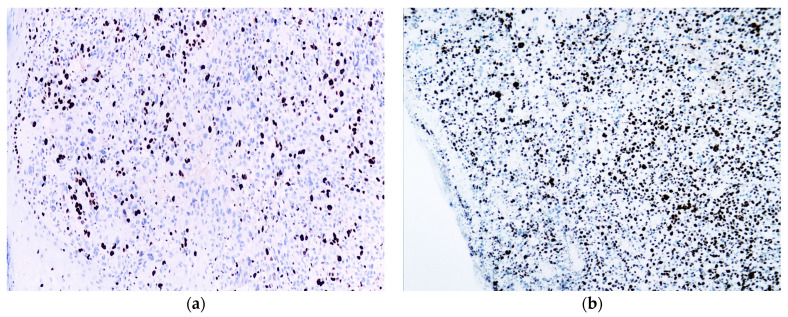
Ki67 positive expression in (**a**) 15% of the tumor cells as the minimum value in the entire group (4×) and in (**b**) 80% of the tumor cells as the maximum value in the entire group (4×).

This analysis highlighted that the proliferation index values are higher in patients with metastatic disease and in those who succumbed to the disease compared to those without disease progression. Overall, within the entire analyzed group, the median Ki67 index was 35%. The minimum value was 15%, and the maximum value of the Ki67 index in the entire analyzed group was 80% (Figure 4).

**Figure 5:**
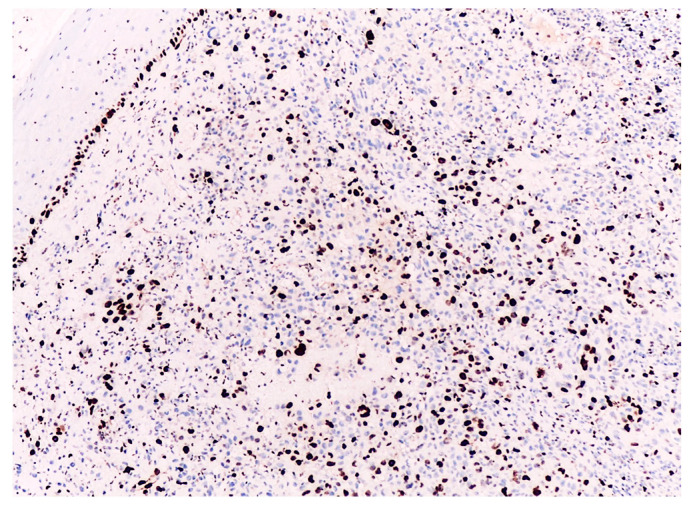
Ki67 positive expression in 30% of the tumor cells as the median value in patients with progression-free survival (4×).

Subsequently, we analyzed the degree of immunohistochemical expression of the Ki67 proliferation index for each subgroup of patients based on disease progression. Starting with the subgroup of patients without progressive disease, we noted that they exhibited a median Ki67 proliferation index of 30% (Figure 5). In this category of patients, the minimum value was 15%, while the maximum value reached 45%.

**Figure 6:**
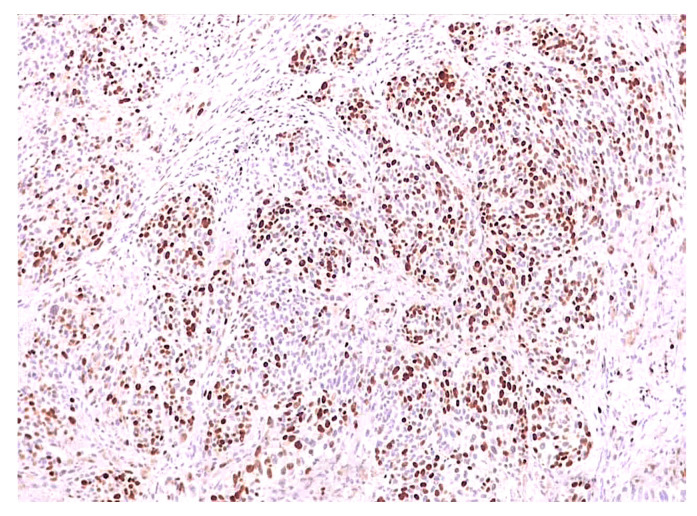
Ki67 positive expression in 60% of the tumor cells as the median value in patients with metastatic disease (4×).

Considering the group of patients who developed metastases during the follow-up, the median Ki67 index was 60% (Figure 6), which is a higher value compared to those without disease progression. However, similar to those without progression, in the case of patients with metastases, the minimum value of the proliferation index expression was 15%, but the maximum value reached 80% within this subgroup.

**Figure 7:**
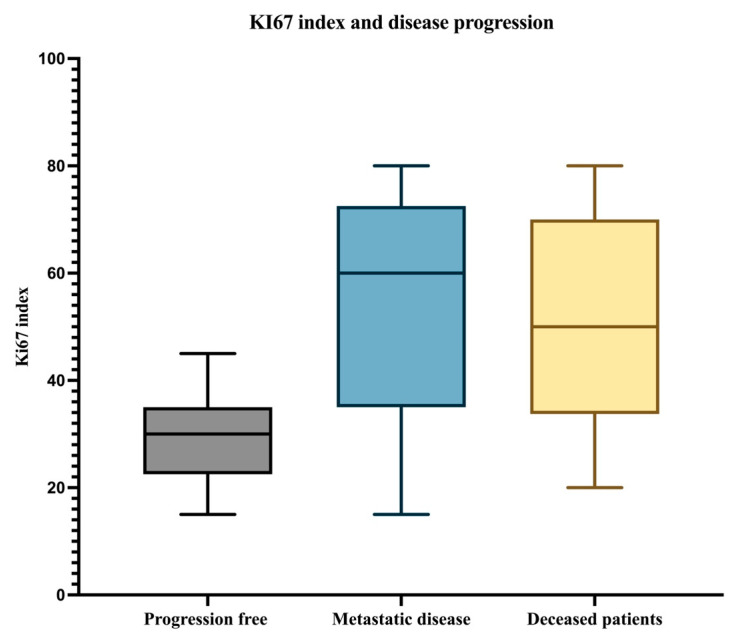
The median, minimum, and maximum values of the Ki67 index in patients with PFS, patients with metastatic disease, and in deceased patients.

In the case of deceased patients, the median Ki67 index was 50%, with a minimum value of 20% and a maximum value of 80%. The median, minimum, and maximum values of the Ki67 index for each subgroup are schematically represented in Figure 7.

Although the median Ki67 index was double in patients with metastatic disease compared to those without disease progression, this difference was not significant in the univariate Cox analysis. However, the Ki67 index was associated with the overall survival of patients (Table 2).

**Figure 8:**
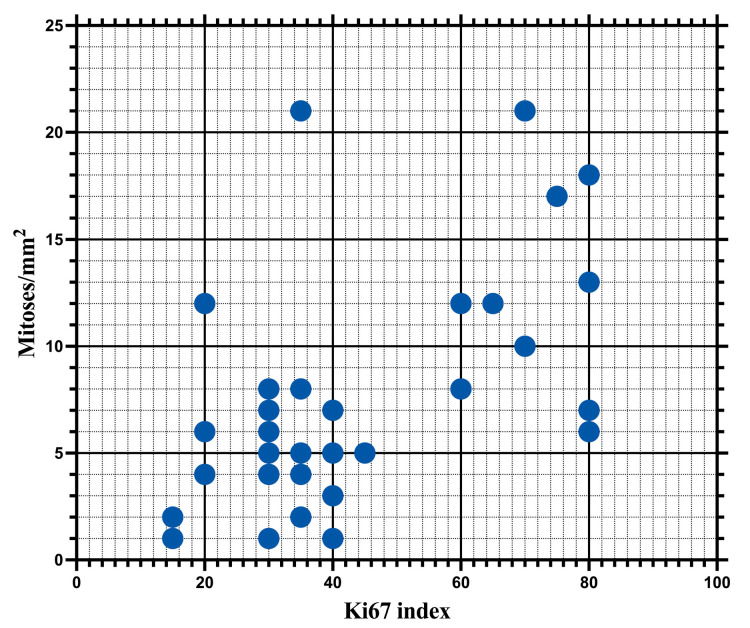
Ki67 index and mitotic counts are strongly correlated (r = 0.5439; *p* = 0.0011).

Considering that both the Ki67 index and the mitotic index were significantly associated with the survival of patients, but not with disease-free interval, we analyzed the degree of correlation between these two parameters. This analysis demonstrated a highly significant statistical correlation (*p* = 0.0011), with a Spearman correlation coefficient of r = 0.5439 and a 95%CI ranging from 0.2366 to 0.7522 (Figure 8).

The next step of this study was assessing the other histopathological parameters conventionally associated with outcomes in cutaneous melanomas (Table 3).

According to the data presented in Table 3, the presence of ulceration, microsatellites, necrosis, lympho-vascular invasion, perineural invasion, and the acral lentiginous and nodular melanoma subtypes are more frequently encountered in patients with metastatic disease and deceased patients. On the contrary, the presence of regression and the superficial spreading melanoma subtype were more often present in the group without progressive disease.

Later on, we evaluated two parameters assessing proliferative activity, along with the other clinical and histopathological characteristics in multivariate analyses for disease-free survival and overall survival (Table 4).

As shown in Table 4, the male gender, Breslow depth, mitotic counts, and perineural invasion were associated with decreased progression-free survival, and age, the male gender, Breslow depth, mitotic counts, and perineural invasion were associated with decreased overall survival. Importantly, the mitotic index was associated with both the disease-free interval and overall survival of patients, while the Ki67 index values no longer had statistical significance.

**Figure 9:**
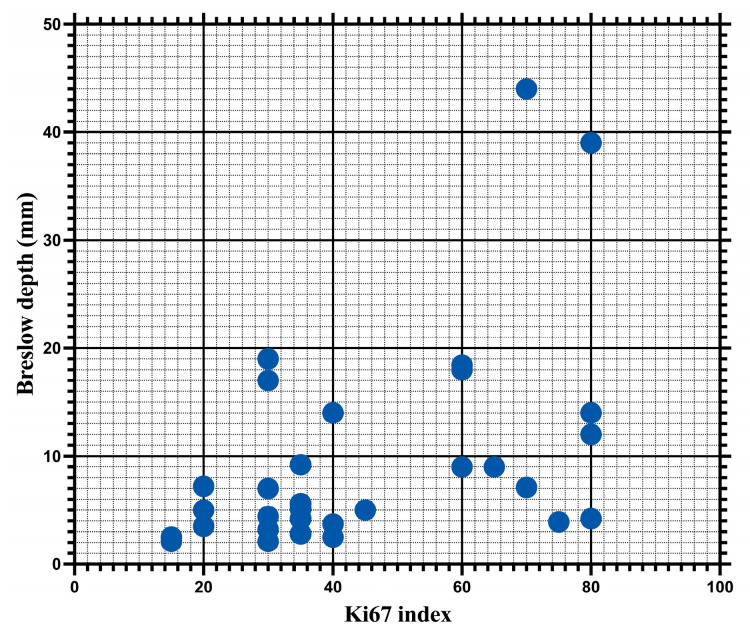
Ki67 index and Breslow depth are positively correlated (r = 0.4749; *p* = 0.0052).

Considering that the invasion depth was strongly associated with both disease-free survival and the overall survival of patients, we evaluated the degree of correlation between the Breslow index and the Ki67 index, as well as the number of mitoses. In this context, the Ki67 index was found to be significantly correlated with the Breslow index (*p* = 0.0052), with a Spearman correlation coefficient of 0.4749 and a 95% CI in the range of 0.1468–0.7088 (Figure 9).

**Figure 10:**
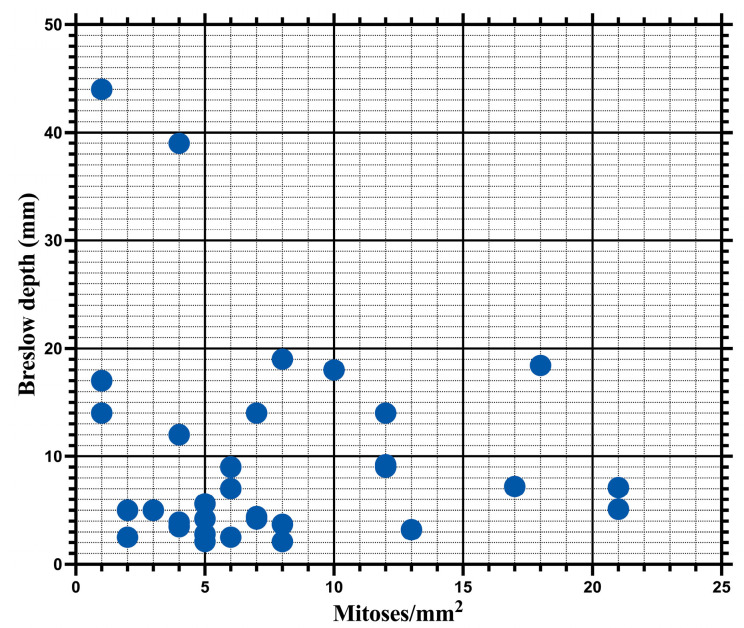
The mitotic rate is not significantly correlated with the depth of invasion (r = 0.01587; *p* = 0.9301).

Unlike the Ki67 index, the number of mitoses was not correlated with the Breslow index (*p* = 0.9301), with a Spearman correlation coefficient of 0.01587 and a 95%CI ranging from −0.3386 to 0.3664 (Figure 10).

**Figure 11:**
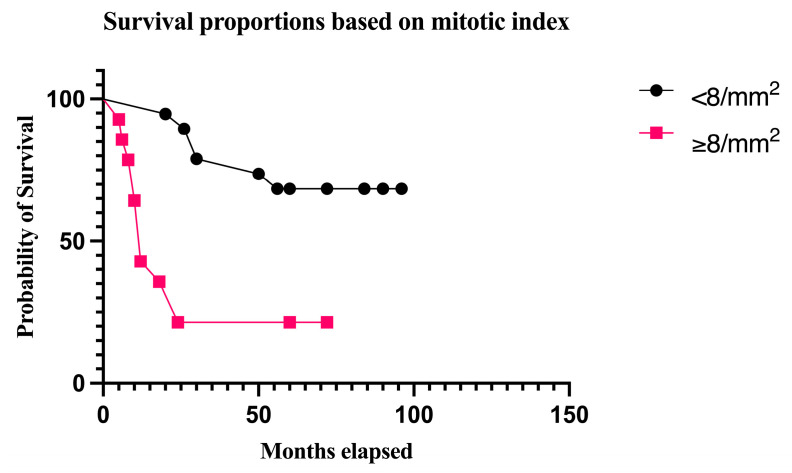
OS stratified by mitotic rate. The survival rate was 68.42% in patients with a mitotic rate of <8/mm^2^. The survival rates decreased to 21.42% in patients with an invasive mitotic rate ≥8/mm^2^ (*p* = 0.0003, log-rank test).

Finally, the prognostic value of mitotic counts and the Ki67 proliferation rate were evaluated by analyzing how these parameters can predict survival rates. The patients were followed-up with for 48.33 months on average (median = 56; minimum = 5; maximum = 96). In this context, as the median mitotic rate in patients who died during the follow-up was 8/mm^2^, this value was used as a cut-off for a survival analysis. The median survival in the group with <8 mitoses/mm^2^ was 72 months, and 6 out of 19 patients in this category succumbed to the disease. On the other hand, the median survival in the group with ≥8 mitoses/mm^2^ was 12 months, with 11 deaths out of 14 patients in this category (*p* = 0.0003, log-rank test) (Figure 11).

**Figure 12:**
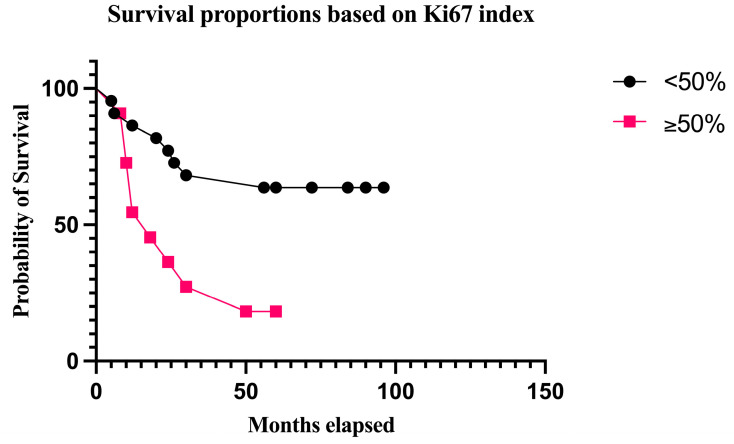
OS stratified by Ki67 index. The survival rate was 63.63% in patients with Ki67 < 50%. The survival rates decreased to 18.18% in patients with Ki67 ≥50% (*p* = 0.0072, log-rank test).

A survival analysis for Ki67 was performed using the 50% cut-off-value as this was the median Ki67 rate in the group of deceased patients. Identical to the group with <8 mitoses/mm^2^, the median survival in the group with Ki67 < 50% was also 72 months, but this category encompassed 22 patients out of which 8 died. The median survival in the group with Ki67 ≥ 50% was 18 months, with 9 deaths out of 11 patients in this category (*p* = 0.0072, log-rank test) (Figure 12).

## 4. Discussion

In the multivariate analysis, the male gender, Breslow depth, mitotic counts, and perineural invasion were associated with decreased progression-free survival and decreased overall survival. In addition to these factors, an advanced age was also associated with OS.

Regarding the prognostic value of the demographic characteristics, there are conflicting reports. Similar to our findings, the male gender has previously been linked with decreased survival by some authors [3,24], while others found no such association [25,26]. Interestingly, an advanced age was only associated with a decreased OS but not PFS, which may be explained by the fact that older patients could have higher mortality rates due to other comorbidities that were not accounted for [27].

Concerning the histopathological factors, our study highlights the prognostic importance of a frequently overlooked parameter in cutaneous melanomas—perineural invasion. Perineural invasion is generally considered unusual and of little prognostic value in cutaneous melanomas, with the notable exception of desmoplastic melanomas where it has been associated with local recurrence [28]. Nevertheless, perineural invasion has also been linked to local recurrence, sentinel lymph node positivity, in-transit metastases, and reduced PFS and OS in other types of cutaneous melanomas [29,30,31,32,33]. Moreover, a few recent studies have proven that perineural invasion is an independent prognostic factor for cutaneous melanomas in general [34,35]. Therefore, recognizing this feature is of utmost importance both for its predictive value and because it could directly influence the therapeutic strategy, with post-operative radiotherapy being a potential for such patients [36].

Interestingly, some well-established prognostic factors such as ulceration, microsatellites, and lymphovascular invasion were not independently associated with any outcomes even though they were more often reported in patients with progressive disease. The lack of association in such cases may be explained by the relatively low number of cases. Nevertheless, a lack of association between these characteristics and disease evolution has been described, particularly in thicker lesions, such as the ones included in this study [35,37].

The mitotic index was also independently associated with both the disease-free interval and the survival of patients. The independent prognostic value of the mitotic rate has been described in various studies [7,29,38,39,40,41]. Despite these results, there is still no consensus regarding the prognostic cut-off value for the number of mitoses as different studies have used various thresholds, such as absent vs. present, ≥1/mm^2^, ≥2/mm^2^, or 5 mitoses/mm^2^ [1,42,43,44,45]. Moreover, the significant cut-off values may vary based on the tumor stage, and further studies are required to fully establish how the mitotic index should be reported in cutaneous melanomas [7,40,46]. Another possible limitation of assessing the mitotic index is the interobserver variability. However, this could be solved by conducting an immunohistochemical analysis of phosphohistone H3 (PHH3). In this instance, PHH3 immunohistochemistry has been shown to improve interobserver agreement and to accurately predict prognosis in various tumors, such as meningiomas and oral epithelial dysplasia [47,48]. Furthermore, this analysis also improves the interobserver agreement in thin cutaneous melanomas [49]. Based on these findings, further studies should be carried out in order to address the sensitivity of PHH3 analysis for more advanced cutaneous melanomas as well as to establish whether this test can improve prognostic estimation.

In the present study, the Ki67 index values were higher in patients with progressive disease and deceased patients compared to those without disease progression. A univariate analysis demonstrated that this parameter is significantly associated with a decreased OS but not with PFS. However, the multivariate analysis did not show any association between Ki67 expression and patient prognosis. When reviewing the existing data in the literature, the utility of analyzing the proliferation marker Ki67 to estimate the prognosis of cutaneous melanomas remains a subject of intense debate. In this context, some studies indicate that the Ki67 index is not associated with PFS or OS in cutaneous melanomas [19]. On the other hand, there are studies that demonstrate a significant association between the Ki67 index and patient prognosis [50]. A meta-analysis from 2021 concluded that the Ki67 index is associated with patient survival but not with the disease-free interval [51]. Parra O. et al. demonstrated that the Ki67 index is independently associated with the survival of patients with cutaneous melanomas [52]. Similarly, Du Y. et al. showed that the Ki67 index is an independent prognostic factor for the survival of patients with acral melanomas. However, in this study, the Ki67 index was analyzed as a dichotomous variable (Ki67 < 30%, Ki67 > 30%) and not as a continuous one [53]. Additionally, in various other studies that have shown an association between prognosis and the Ki67 index, it was analyzed as a dichotomous variable with different threshold values [21,54,55,56,57]. In our study, the Ki67 index was analyzed as a continuous variable, with 28 out of the 33 tumors analyzed showing a Ki67 index above 30%. These reports highlight the need for establishing a standardized threshold value for evaluating cutaneous melanomas. We believe that in the absence of a consensus regarding a threshold value for Ki67, it should be analyzed as a continuous variable. This method of analyzing the proliferation index should be especially considered in cases of melanomas with an invasion depth over 4 mm, as these typically exhibit increased Ki67 index values. Alternatively, similar to the mitotic rate, different Ki67 cut-off values for each tumor stage may be beneficial for predicting prognosis in cutaneous melanomas.

Equally, we recognize the need to establish a possible correlation between the Ki67 index and invasion depth. From this perspective, in this study, we demonstrated a statistically significant correlation between the Ki67 index values and those of the Breslow index. These results are supported by a meta-analysis that highlighted that the Ki67 index is correlated with the invasion depth, being significantly higher in melanomas with a Breslow index of over 4 mm [51]. Furthermore, various other authors have observed that the Ki67 index is correlated with the invasion depth, showing that, similar to our study, this marker is not independently associated with patient prognosis [10,21,54,55]. On the other hand, the mitotic index, unlike the Ki67 index, was not significantly correlated with the invasion depth. These results may seem surprising as most authors report a positive correlation between the mitotic rate and Breslow index [37,39]. The lack of correlation in our study may be explained by the fact that only pT3 and pT4 tumors were included.

Finally, another disadvantage of Ki67 in estimating cutaneous melanoma prognosis is the lack of a standardized method for evaluating this index. Depending on the study, there may be significant variations in the analysis approach [10,18,19,21], further complicating the establishment of a threshold value with prognostic utility for cutaneous melanomas. The method we proposed in this paper has shown a good inter-observer level of agreement. Nevertheless, the method used in this paper is a tedious process which can limit its usefulness on a larger scale. Another possible limitation of Ki67 analysis is that this marker can also stain intratumoral lymphocytes, thus overestimating the proliferation rate. This shortcoming could be solved by using a double stain such as Melan-A/Ki67 or HMB45/Ki67. The cytoplasmic positivity of Melan-A and HMB45 encountered only in melanoma cells allows for a correct counting of Ki67 only in the tumor proliferation [58].

Concerning potential predictive cut-off values for both the mitotic rate and Ki67 index, we performed survival analyses using the median values in the group of deceased patients as thresholds. Consequently, a mitotic rate ≥8/mm^2^ and Ki67 ≥ 50% were strongly correlated with decreased survival in the Kaplan–Meier analyses. Both variables demonstrated similar median survival times and survival rates, which once more supports the correlation between mitotic figures and the Ki67 index. However, the statistical significance was stronger for the survival analysis based on the number of mitoses.

For all of these reasons, despite the promising results, we believe that further studies are needed to establish a threshold value with prognostic significance as well as a standard for quantifying the Ki67 index before integrating this variable into the assessment algorithm for the prognosis of cutaneous melanomas. At present, the additional time and financial resources required for performing immunohistochemical tests for Ki67 are not justified as this proliferation marker remains inferior to the mitotic rate in predicting the outcomes of patients with cutaneous melanomas.

## 5. Conclusions

Cutaneous melanomas are aggressive yet unpredictable malignancies that are in urgent need of new prognostic markers that are easily interpreted regardless of examinator experience or available resources. In this context, we demonstrated that perineural invasion is a reliable independent predictor for both PFS and OS. This still infrequently analyzed feature may become an important parameter influencing the clinical management of cutaneous melanomas. Furthermore, the mitotic rate is also an independent prognostic factor, while the Ki67 index does not predict disease evolution independently. Nevertheless, both parameters significantly predicted survival in Kaplan–Meier analyses. However, further studies are required to establish universal cut-off values for both parameters as well as a standardized method for evaluating Ki67 positivity.

## Figures and Tables

**Table 1:** Cox univariate analysis for mitotic index.

	Progression-Free Survival			Overall Survival		
	HR	95%CI	*p*	HR	95%CI	*p*
Mitotic index	1.239	0.95–1.65	0.1049	1.1	1.29–1.66	0.0126

**Table 2:** Cox univariate analysis for Ki67 index.

	Progression-Free Survival			Overall Survival		
	HR	95%CI	*p*	HR	95%CI	*p*
Ki67 index	1.14	1.01–1.36	0.0696	1.1	1.03–1.23	0.0302

**Table 3:** The histopathological characteristics of the tumors based on patient outcomes.

	Entire Group (*n* = 33)	Progression-Free Survival (*n* = 12)	Metastatic Group (*n* = 21)	Deceased Patients (*n* = 17)
Melanoma subtype				
Superficial spreading	30.30% (*n* = 10)	66.67% (*n* = 8)	9.53% (*n* = 2)	11.76% (*n* = 2)
Acral lentiginous	18.18% (*n* = 6)	0	28.57% (*n* = 6)	35.29% (*n* = 6)
Nodular	51.52% (*n* = 17)	33.33% (*n* = 4)	61.9% (*n* = 13)	52.95% (*n* = 9)
Ulceration	72.72% (*n* = 24)	41.66% (*n* = 5)	90.48% (*n* = 19)	94.12% (*n* = 16)
Regression	6.06% (*n* = 2)	8.33% (*n* = 1)	4.76% (*n* = 1)	5.88% (*n* = 1)
Microsatellites	15.15% (*n* = 5)	0	23.81% (*n* = 5)	23.53% (*n* = 4)
Necrosis	48.48% (*n* = 16)	25% (*n* = 3)	61.9% (*n* = 13)	58.82% (*n* = 10)
Lympho-vascular invasion	39.39% (*n* = 13)	25% (*n* = 3)	47.62% (*n* = 10)	17.65% (*n* = 3)
Perineural invasion	24.24% (*n* = 8)	8.33% (*n* = 1)	33.33% (*n* = 7)	17.65% (*n* = 5)

**Table 4:** Cox multivariate analysis for PFS and OS.

	Progression-Free Survival			Overall Survival		
	HR	95%CI	p	HR	95%CI	p
Age	1.038	0.99–1.09	0.0998	1.23	1.08–1.51	0.0147
Gender (male)	5.79	1.40–26.59	0.0171	84.46	5.31–3744	0.0076
Melanoma subtype						
Superficial spreading	Ref					
Acral lentiginous	9.79	0.86–132.2	0.0686	2.48	0.13–75.44	0.5652
Nodular	1.08	0.14–9.71	0.9405	0.08	0.003–1.41	0.0989
Breslow depth	1.08	1.01–1.15	0.0144	1.31	1.13–1.61	0.0018
Ki67 index	0.99	0.96–1.02	0.6727	0.98	0.94–1.02	0.4263
Mitotic index	1.16	1.01–1.33	0.0276	1.31	1.05–1.79	0.0428
Ulceration	0.41	0.05–2.29	0.3421	0.4	0.008–8.07	0.5804
Regression	0.88	0.04–7.079	0.9228	0.61	0.008–18.64	0.7985
Microsatellites	0.55	0.09–2.68	0.9228	0.01	0.0001–0.61	0.058
Necrosis	0.26	0.05–1.13	0.084	0.62	0.08–3.67	0.6032
Lympho-vascular invasion	1.08	0.26–4.56	0.9091	0.09	0.005–1.26	0.075
Perineural invasion	6.45	1.48–30.09	0.0131	35.68	2.8–1065	0.0167

## Data Availability

All of the data processed in this article are part of the research for a doctoral thesis, which is archived in the pathology department at the University Hospital of Bucharest, where the interventions were performed. The original data are available upon reasonable request.
